# Burnout Syndrome in Police Officers and Its Relationship with Physical and Leisure Activities

**DOI:** 10.3390/ijerph17155586

**Published:** 2020-08-03

**Authors:** Blanca Rosa García-Rivera, Jesús Everardo Olguín-Tiznado, Mónica Fernanda Aranibar, María Concepción Ramírez-Barón, Claudia Camargo-Wilson, Juan Andrés López-Barreras, Jorge Luis García-Alcaraz

**Affiliations:** 1 Faculty of Administrative and Social Sciences, Autonomous University of Baja California, Valle Dorado, Ensenada, BC 22890, Mexico; blanca_garcia@uabc.edu.mx (B.R.G.-R.); maranibar@uabc.edu.mx (M.F.A.); cony@uabc.edu.mx (M.C.R.-B.); 2 Faculty of Engineering, Architecture and Design, Autonomous University of Baja California, Ensenada, BC 22860, Mexico; jeol79@uabc.edu.mx (J.E.O.-T.); ccamargo@uabc.edu.mx (C.C.-W.); 3 Faculty of Chemical Sciences and Engineering, Autonomous University of Baja California, Tijuana, BC 20631, Mexico; jalopez@uabc.edu.mx; 4 Department of Industrial Engineering and Manufacturing, Autonomous University of Ciudad Juarez, Ciudad Juarez, CHI 32310, Mexico

**Keywords:** burnout syndrome, fatigue, psychosocial risk factors, work-related exhaustion, police

## Abstract

No previous studies in Mexico have been found that jointly analyze physical and leisure activities as variables related to mental health in police officers. This paper presents research on burnout in Mexican Police officers. The question it answers is: is there any association of burnout with physical and leisure activities and personal profile? A total of 276 police officers (87% men and 13% women) participated. To obtain information, the Spanish Burnout Inventory and the Operational Police Stress questionnaires were used. A cross sectional study design was utilized with tests of validity and reliability, goodness of fit, analysis of variance (ANOVA), and analysis of k-means clusters. Results showed that a high number of policemen had high prevalence of burnout and a high level of mental exhaustion, and that exercise was positively and significantly related to lower burnout risk. Men showed higher risk than women. Results should be considered to improve interventions and occupational health practices in the police force. This paper improves understanding of burnout among policemen and the importance of exercise and leisure activities to alleviate burnout.

## 1. Introduction

Burnout has been a topic of interest for academics and professionals for the last five decades [1,2,3,4] A search in academic databases yielded more than 39,000 articles found on the topic. Academics have defined burnout as a syndrome induced by the job; for example, Freudenberger (1974) first defined it as the observation among workers of disappointment and loss of desire that developed into burnout. Pines et al. (1981) defined it as an emotional demand that physically, mentally and spiritually trespassed on worker limits. Maslach et al. (2001) defined it as a prolonged response to chronic emotional and interpersonal stressors on the job. Schaufeli et al. (2009) defined burnout as a mix of psychological, behavioral and physical symptoms. Later, Grantt et al. (2018) defined it as a chronic stress reaction. Other researcher discussed a negative phenomenon observed in workers caused by mismatch of intentions and reality at the job and inadequate coping strategies. The literature mentions up to 100 symptoms as an indicator of burnout [1,2,3,4,5,6,7,8,9,10,11,12,13,14,15,16,17,18,19,20]. 

Researchers have found that main consequences of burnout include withdrawal from work, overwhelming feelings of exhaustion, ineffectiveness and detachment at the workplace, lack of job involvement and low job accomplishment, physical fatigue, and dehumanization, also known as depersonalization. Impaired work performance as a consequence because of negative attitudes and behaviors is also mentioned. This syndrome develops feelings of exhaustion, cynicism, and a personal lack of accomplishment according to Maslach et al. [11]. 

To identify and find new evidence relating to burnout is of great importance for organizations to prevent further negative consequences.

Nowadays, the interest in this topic has grown all over the world since evidence shows that it is associated with negative job consequences such as absenteeism, presentism, turnover, negative health outcomes such as insomnia and sleep disturbances, depression, anxiety, impaired job performance, fatigue, irritability and frustration, cynical attitudes, professional inefficacy, depersonalization, neglect, withdrawal from work, diminished professional efficacy and poor quality of life [21,22,23]. In Mexico, labor regulations recently stablished psychosocial factors, including burnout, as a job risk that should be prevented by organizations, or high penalties could be applied. 

The literature on burnout and Police officers has been extensive, associating this profession with a high risk of burnout as a negative long-term consequence of the job [24,25,26,27,28]. Police officers are a group particularly vulnerable to psychopathological disturbances, which leads them to suffer from burnout and affects their quality of life [21,27]. Previous studies show that police officers involved in life-threatening situations, extreme violence, critical incidents, shootings, hostage or negotiation situations, intense exposure to crime scenes, or coworkers’ death or suicide, can develop burnout syndrome associated with mental health problems [5,24,29,30,31,32]. 

One of the most stressful professions is police work [33] in which evidence shows that high levels of continuous stress lead to burnout [28,33]. Stress related to work activity has become a pattern of reactions, of psychological, emotional, cognitive, and behavior types, that directly relate to specific characteristics linked to job performance [5]. Psychological consequences can be acute or chronic, and they can either appear or return when a new event is perceived as traumatic and/or no organizational support is perceived, as shown in the Demand Control Social Support Model [34]. 

Despite the fact that numerous studies have been conducted on the analysis of burnout in police officers worldwide [31,33,35,36,37], in Mexico there are very few studies related to police officers’ psychopathologies that affect their performance, how these affects relate to their particular conditions and how they correlate with their personal profile and life style related to physical and recreational activities, even more so considering that now there is an obligation for institutions to watch for employees’ psychosocial risks as the new regulation is enforced in Mexican law. Previous studies show that relationships between physical activity, stress, and burnout have received limited attention. Moreover, studies have shown inconsistent results concerning the relationship of physical activity and burnout [38]. On the other hand, occupational stress and burnout in women have had very little research attention. There is an important need to conduct research in this area, since police women are facing dual careers in balancing home and work related issues [39]. Finally, we can state that no previous studies in Mexico have been found that jointly analyze these variables related to mental health in police officers; therefore, it is of high importance to address this subject.

Implications can be of high importance in preventing further consequences. Previous studies of police officers have found a strong association between psychosocial risks and job pathologies derived from burnout [40,41,42]. Furthermore, studies conducted into the police forces in Mexico and Latin America have always focused on analyzing corruption and lack of values, leaving aside the detection of work-related psychosocial factors that affect police officers and lifestyle activities that mitigate this condition [43,44]. 

### 1.1. The Nature of Police Work in Mexico

The population of the State Police in Baja California is 3800 officers including administrative employees and preventive police officers. From this figure, 844 active officers belong to the Municipal Police in Ensenada, where the study was performed. From this number, 154 police officers retired and only 30 new officers were recruited in 2018 in Ensenada, this according to information from the Administrative Office of the Municipality. In addition, in the same year, three municipal police officers were killed. Eleven others were released from their positions due to failing the control and trust examinations, another for absenteeism, another for working under the influence of alcohol, and one officer is currently suspended after being arrested in the United States on charges of drug trafficking, according to reports from the public prosecutor’s office.

At this date, active police officers in Ensenada is *n* = 696. Analyzing this number, there should be 300 officers per every 100,000 inhabitants (according to indicators of the Citizens’ Council of Ensenada), and if the population of Ensenada is estimated to be half a million, there should be at least 1500 active police officers.

When analyzing the number of active police officers currently available, it is observed that most suffer from addictions and severe post-traumatic stress due to the high criminal rates they are facing and the acute deficit of officers in the municipality. In these conditions, a large number are exposed to psychosocial work issues and emotional exhaustion. Adding to this situation, more than 1280 murders were reported in the state last year and it is considered one of the states with the highest incidence of murders all over the country. Police forces’ precarious budgets, low wages and lack of social security add to this situation [45]. 

The State Police Force faces high turnover and poor “team spirit” based on poor solidarity and group cohesion, poor social recognition of the value of the professional function and merit, and lack of institutional resources for effective and sufficient protection [46]. A lack of resources at times of intense pressure, such as time or human and material resources, are associated with mental or physical health issues [25,31,47]. The fact that citizens consider that the police force is incapable of solving the insecurity problems of the country and do not trust them, thinking of police as “weak and associated with crime”, also aggravates pressure and chronic stress leading to burnout [33,46]. Today, the fight between drug lords has created a hostile environment where there are daily deaths with excess cruelty and violence, killing police officers who do not cooperate with their business or who oppose their deals [45]. This entire context in which police officers carry out their job affects them severely, stresses them out, and leads them to psychosocial risks where drug use and alcoholism are common; hence, the importance of studies and programs that pay attention to this problem.

#### Criteria of Inclusion of Physical and Leisure Activities

The McCreary questionnaire includes an item that mentions “*finding time to stay in good physical condition*”. It is not specific and it does not include minimum time or type of exercise. This is a question that allows the policemen to reply if they feel they are in good shape due to exercise. Other studies using the McCreary questionnaire mention that the different types of physical activity and treatment modalities used in the included studies impede clear conclusions. However, sport or exercise type as long as it is regular does not seem to be decisive for the effectiveness of the exercise-based therapy. For leisure activities, the purpose was to make friends outside the job and to extend their social life. Like physical activity, leisure type does not seem to be decisive for effectiveness as long as the intention of making friends and having a social life is fulfilled [48,49].

### 1.2. Physical and Leisure Activities and Burnout

The relationship between stress, burnout and healthy activities has been a subject of interest for academics and professionals over the years. Important findings have shown consistent association between them; the higher stress and burnout, the poorer physical and mental health will become. Adverse health symptoms related to burnout are affecting the quality of service of Policemen [50]. Strong evidence for the effect of activity on reducing exhaustion in intervention studies has shown the importance of physical and leisure activities as an effective way to reduce burnout [51]. Hence, the need for studies to analyze the influence of physical and leisure activities on burnout [48,50,52]. Evidence has shown that regular physical activity facilitates psychological detachment from work, and reduces the risk of prolonged stress responses such as burnout [53]. Findings suggest that “exercise induce changes resulting in better mood and increased energy” [51,54,55]. 

### 1.3. Hypotheses

The aim of this study was to examine the relationship between burnout, physical, leisure activities and personal profile in Mexican police officers. The following hypotheses have been formulated:

**Hypothesis 1** **(H1).** *Significant differences exist in the prevalence of burnout syndrome between police officers who engage in physical activity and those who do not*.

Evidence shows that physical activity has a significant negative correlation with burnout. Exercise programs change the cause-effect relationship between burnout and metabolic syndrome components, improving mental health and physical wellbeing [42]. A well-designed exercise program establishes better health behavior in the workplace by alleviating job strain factors and metabolic syndrome components [11,49,56,57]. Exercise causes a positive impact on mental health and there is a strong positive association between physical activities and wellbeing [56].

**Hypothesis 2** **(H2).** *Significant differences exist in the prevalence of burnout syndrome between police officers who engage in leisure activity and those who do not*.

Evidence shows that leisure activity has a significant negative correlation with burnout. Organizational encouragement of team members to participate in leisure activity promotes workplace involvement, motivation, provides social opportunities for employees to interact and has a positive impact on mental health [10,58,59].

**Hypothesis 3** **(H3).** *Significant differences exist in the prevalence of burnout syndrome between police officers with stable marriages and single officers*.

Evidence shows that single individuals present higher emotional exhaustion and depersonalization than married ones and show lower personal achievement [60]. Single officers experience burnout more than the married [41]. Singles may require help and organizational support in dealing with burnout [10,60].

**Hypothesis 4** **(H4).** *Significant differences exist in the prevalence of burnout syndrome between male police officers and female police officers*.

Evidence shows that women present higher work related stress and burnout than man due to their double roles [61]. Women are prone to a higher prevalence of burnout due to their responsibilities and traditional roles as mothers and wives [39,62,63] and job stress tended to result in higher level of occupational burnout in female than in male police personnel [64].

**Hypothesis 5** **(H5).** *Significant differences exist in the prevalence of burnout syndrome between police officers with daily operational stressors and police officers with occasional operational stressors*.

Evidence shows that stress and burnout perceived by police officers that work in areas of extreme violence is higher [65]. Individuals within facilities benefit from a healthier workforce since they experience less operational stressors [66]. 

## 2. Materials and Methods

### 2.1. Sample Calculation

This study used primary data collected from police officers from the Municipal Police Force of Ensenada, Mexico. The population of policemen in Ensenada consisted of 720 officers. A census was applied to the total population. Questionnaires were delivered and 276 were completed and returned. This represents 38.33% of the total. 

### 2.2. Participants

The police headquarters were used for the application of the survey, requesting authorization from high-ranking officers. Participation was voluntary for all police officers. The complete workforce was invited to participate. 

### 2.3. Measures and Instruments

For the purpose of measuring burnout, the (SBI) Spanish Burnout Inventory [67] was used. We decided to use this questionnaire after an extensive analysis of its psychometric properties published in a previous paper where we made a meta-analysis of the properties of the SBI, finding that for Latin American countries results indicated that the SBI possessed adequate psychometric properties for the study of burnout compared to other questionnaires such as the MBI (Maslach Burnout Inventory) that contain content and psychometric problems which suffer when translated into Spanish [67,68]. 

The SBI (Spanish Burnout Inventory) questionnaire was developed by Gil-Monte et al. [67] and Gil-Monte and Noyola Cortés [69]. This questionnaire is used widely by Spanish speaking countries with better results than the MBI. The dimensions of the SBI are: (a) Enthusiasm towards the job, (b) Emotional exhaustion, (c) Indolence, and (d) Guilt [67]. The SBI is built of 20 items valued with a Likert-type response system. It evaluates through a range of 5 adjectives, ranging from “never” to “every day”, the frequency of the situations perceived. Low scores in enthusiasm towards the job and high scores in indolence, guilt, and emotional exhaustion would indicate the presence of burnout. The instrument has been applied to health and education professionals, police officers, and prison guards.

The McCreary & Thompson’s questionnaire (PSQ-OP, 2006) was also used to measure operational stressors of professional activity in police officers. Only the first nine items of this instrument were used due to their relation to the job elements. The rest of the items such as eating healthy, higher image, stigma, family, etc., were not related to this research and we decided not to include them. Items were evaluated with a Likert scale of 7 options ranging from “never” to “every day”. The questionnaire consists of 20 items that analyze indicators such as favoritism, administrative work, lack of resources, inequality in the workload, obsolete equipment, and their relation to the stress they cause. 

### 2.4. Data Analysis

The information was entered into a database created in the SPSS software v.24 (IBM, Armonk, NY, USA), where the rows represented each case and the columns represented the items in the questionnaires. For the cleansing of the database, the following was performed:

- The lost values were replaced by the mean in cases where the percentage was less than 10% and, if it was higher, that case was eliminated.
- Extreme values were identified through the standardization of each item. Absolute values higher than four are considered extreme values and were replaced by the mean of the item.

### 2.5. Procedure

This was a cross sectional design. The study analysis procedure was conducted in two moments. The first was validity analysis of the instrument to identify predictive capacity. In the second moment, a results’ analysis was conducted to evaluate the conditions of prevalence of burnout and the relation with personal profile, recreational activities, and physical exercise, thus validating the hypotheses suggested.

A descriptive analysis of the information was conducted in which the sample mean, the standard deviation, and Cronbach’s alpha were obtained for each analyzed dimension. The higher the score obtained in the items (close to five), the higher the prevalence of the syndrome, whereas a lower score lowers the prevalence of the syndrome.

For the validity analysis of the instrument, an analysis of the Spearman’s correlations, Cronbach’s alpha and exploratory factor analysis was conducted with the aim of validating the conditions of convergence, divergence, and the reliability of the instrument.

Likewise, for the results’ analysis, an analysis of k-means clusters was conducted under the nearest neighbor method with the purpose of identifying the determining attributes that could generate persistence of burnout syndrome. Additionally, an analysis of variance (ANOVA) was conducted with the purpose of identifying how after-work activities (recreational activities), demographic conditions (marital status and gender), and work conditions (working hours) can decrease or generate the prevalence of the syndrome. Normality of random errors, homogeneity of variances and compound symmetry tests were performed.

### 2.6. Ethics Statement

This study was performed accordingly to the written informed consent from all policemen participants. The protocol was approved by the Postgraduate Department Committee of the Faculty of Administrative and Social Sciences of Universidad Autonoma de Baja California, according to the NOM-035-STPS-2018, the ethic statement was reviewed and approved.

## 3. Results

### 3.1. Descriptive Analysis of the Sample

The questionnaire was applied to 276 preventive police officers in Baja California (87% men and 13% women). 82% have a high school education, the remaining 18% have a bachelor’s degree, and their ages fluctuate from 23 to 42 years old. 58.7% have a seniority of 6 to 10 years, 34.8% of 2 to 5 years, and the rest of 11 to 15 years. All were volunteers in the sampling.

From the total sample, 3% work in operational areas and the rest in technical areas. Of the total, 60.9% of them are married, 26.1% single, and 13% live in civil union. 50% mentioned working 60 h a week, whereas only 32.6% stated working 48 h a week. All work to a mixed schedule. A total of 43.5% generally experience shift changes, whereas 19.6% only work the night shift. A total of 34.8% mentioned usually working overtime and 26% claimed to always work on their days off. Regarding their safety, 32.4% mentioned usually feeling at risk of being injured in their job and 13% stated feeling that way very often. They also mentioned the lack of protective gear since they are not given bulletproof vests; they must buy them themselves.

A total of 43.4% mentioned handling traumatic events such as shootings, clashes, deaths, and wounded people very often. Regarding health, 23% were suffering from gastritis, 21.7% from back pain, 6.5% from insomnia and neck pain, and 2.2% suffer food-related anxiety. A total of 52.2% do not engage in any sport, 73.9% do not practice any recreational activity, and 52% stated that any personal life outside work does not exist.

### 3.2. Descriptive Analysis of the Items

In Table 1, a summary of the questionnaire’s items is observed. Additionally, results obtained by Figueiredo-Ferraz, et al. [69,70] are shown for comparative purposes. Specifically, in the dimension of work satisfaction it is showed that aspects related to personal self-realization and job gratification are well-valued conditions of professional work, which places these conditions as protective aspects against the possible risk of burnout syndrome.

Regarding the dimension of mental exhaustion, aspects related to personal work over-saturation, the existence of burden-related feelings, physical exhaustion, and emotional exhaustion are conditions observed in 39% of respondents. This condition is similar to attributes of the dimensions of guilt and indolence, in which those aspects related to concern for working with people, to the existence of guilt-related feelings and remorse, to the absence of attention and the indifferent and ironic treatment of those requiring attention, are conditions that are present in the professional work of the studied sample.

By comparing the scores in this study with other research that used the same questionnaire, it is possible to notice that the studied sample presents on average lower levels of the prevalence of burnout, considering that, in the case of teachers in the Mexican context, the dimension of mental exhaustion is more frequent [69] and in the case of police officers in the Portuguese context, the dimension of work excitement shows less frequently [70].

Regarding asymmetry values, their absence is recognized, taking into account that it shows values of less than one in (A ≤ |1|) absolute value; this condition corroborates the normality of the scale, which is necessary for the analyses that are shown in this study. In light of this, it is important to highlight that, in the studies with which the scores of the burnout scale were compared, in most of the items the absence of a scale of normality is shown, which ostensibly affects the expected results.

### 3.3. Analysis of Clusters

From the analysis of k-means clusters via the criterion of the nearest neighbor, it is possible to identify two sample groups.

The first group consists of 45% of police officers and is characterized by regularly presenting the operational processes of their professional activity: shift changes, working night shifts, request to work overtime, risk of being injured at work, working on days off, handling traumatic events, not having time to spend with family and friends, and a lot of paperwork for the performance of their tasks. This cluster was named ‘cluster with daily operational stressors’. Likewise, the second group consists of 47% of police officers and is characterized by occasionally presenting attributes relating to operational processes of their professional activity. This cluster was named ‘cluster with occasional operational stressors’.

Furthermore, from the k-means analysis using the Euclidean distance via the method of the nearest neighbor, it is recognized as a relevant aspect that each of the attributes have highly significant differences (*p* value ≤ 0.01) between the two identified clusters. This shows that the changes in the level of stress perceived by operational activities carried out during professional work by the studied subjects are directly due to multidimensional conditions that are not manifested individually, but conversely. For a variation of the stress perceived to occur, adverse operational conditions must be presented jointly (see Table 2).

Likewise, an analysis of k-means clusters was conducted using Euclidean distance via the criterion of the nearest neighbor with the aim of identifying groups of subjects who present, among the attributes of burnout syndrome, highly significant differences (*p* value ≤ 0.05). Here, two sample groups are identified. The first consists of 60% of the police officers surveyed, who perceive work as a source of personal realization, gratification and excitement, and as a challenge. In addition, they almost never feel swamped, overwhelmed with physical and emotional exhaustion or with guilt-related feelings, and they rarely assist citizens in an indifferent, ironic, or mean way. For this reason, this group was named ‘cluster with absence of burnout syndrome’. The second group consisted of the remaining 40% of police officers. This was characterized by almost never perceiving work as a source of personal realization, gratification, excitement, or as a challenge. Members of this group feel swamped, overwhelmed, physically and emotionally exhausted with guilt-related feelings, and tend to treat citizens in an indifferent, ironic or mean way. For this reason, it was named ‘cluster with prevalence of burnout syndrome’.

Furthermore, from the k-means analysis using Euclidean distance via the method of the nearest neighbor, it is recognized as a relevant aspect that not all of the attributes have highly significant differences (*p* value ≤ 0.05) between the two identified clusters. This shows that the attributes that generate prevalence of burnout syndrome are: not to see work as gratification, not to see work in a positive way, to feel swamped or overwhelmed with physical and emotional exhaustion, to have feelings of guilt and remorse regarding work-related attitudes, not to assist citizens who require service, and to assist them with indifference or in a mean, ironic and disrespectful way (see Table 3).

### 3.4. Anova

Lastly, the analysis of variance (ANOVA) (see Table 4) was conducted in order to identify statistically significant differences in the prevalence of burnout syndrome between professionals who engage in sport activities and those who do not, professionals who practice recreational activities, and professionals in a stable marriage and those who are single, as well as male and female professionals, and professionals with daily operational stressors compared to professionals with occasional operational stressors.

In Table 4, it is stated that marital status, whether or not one has a stable partner, and the development of recreational activities do not show significant differences in the prevalence of burnout syndrome (*p* value ≥ 0.05); whereas engaging and not engaging in sport activities show significant differences in the dimension of mental exhaustion (*p* value of ≤ 0.05). There are also significant differences between men and women in the dimension of mental exhaustion (*p* value ≤ 0.05); and there are significant differences between the cluster with occasional operational stressors and the cluster with daily operational stressors in the dimensions of mental exhaustion and guilt (*p* value ≤ 0.05).

The above allows us to accept the first hypothesis that there are significant differences in the prevalence of burnout syndrome between professionals who engage in sport activities and those who do not; hypothesis four which states that there are significant differences in the prevalence of burnout syndrome between professional men and women; and hypothesis five which states that there are significant differences between professionals with daily operational stressors and professionals with occasional operational stressors. It is emphasized that these are accepted, considering that the hypotheses were not comparing the totality of the dimensions of the burnout syndrome, only the dimension of mental exhaustion for each of the groups to be contrasted, plus the scale of guilt for the last group. Below, we find Table 5, where a summary of the results of the hypotheses stated in this investigation is made:

## 4. Discussion

The aim of this investigation was to identify the prevalence of burnout in 276 State Police officers in Baja California, and its relationship with personal profile. The percentage of police officers who show warning signs of burnout syndrome was 40%, considering that these have a lower level of work excitement and a higher level of mental exhaustion. This proportion is very similar to what has been found in other professions in Mexico, as in physicians in Guadalajara with a percentage of 42.3% [71]; in police officers or prison staff in Spain, at 43.6% [70]; and in Costa Rican police officers, at 44.4% [72].

This study has helped to understand the effects of burnout in a sample of police officers. Preventive measures must aim to improve the quality of life of this sector of employees, who are vulnerable not only in their safety but also in the protection of their mental and physical health. Burnout is a silent enemy that affects the whole organization, reduces performance and the quality of service, and prevents staff from performing with efficiency. The practice of sport and recreational activities, as observed, must be a priority for police officers on a daily basis due to the moderating effect of these activities and their feature as a stress and burnout reducer. This should also be considered as an intervention method for police officers that present higher levels of burnout. Prevention must be aimed at addressing the structure of sport and individual leisure activities in order to diminish exposure to stress, promote autonomy and support and increase coping resources and strategies. Taken together, we hope that these findings will enable police head supervisors to better recognize burnout and its risk factors and help prevent its development.

In Mexico, although a regulation was recently established in the Federal Labor Law that recognizes burnout as a psychosocial disease, there have yet to be measures put in place to monitor the condition of the policemen who suffer burnout. Likewise, it is recommended that a program is implemented that provides the necessary support to police officers, since it is observed that this phenomenon is increasingly common among these professionals. Also, rehabilitation programs should be implemented for those with problems with addictive substances, and psychological support programs to improve their quality of life in the face of the post-traumatic stress they suffer.

The theoretical implications of these findings have similarities with other studies conducted in European countries where the results of Figueiredo-Ferraz et al. [70] and Gil-Monte and Noyola Cortés [69] show similar but higher values for measuring police populations. Turgoose et al. [73] also show similar results regarding burnout in police officers with high levels of violence and secondary trauma. This highlights the cultural aspects of Mexico, as Losada and Otero [74] mention, who showed that the sense of humor that characterizes Latinos is a resilient feature that reduces negative emotional burden and, therefore, burnout levels. The construct of a sense of humor allows police officers to feel stronger in the face of adverse circumstances; it allows them to adapt, to laugh at oneself, and to focus on reality with a greater tolerance [74].

We can conclude that organizations and public institutions in Mexico need to pay increasing attention to the organizational behavior of employees. As long as institutions develop a perspective on employee well-being as a means for the institution to improve quality of service and productivity, we will start to see changes such as improved engagement, less exhaustion, and happier employees that use physical activity and leisure activities as part of their daily routine to keep a positive mind set and avoid medication and drug addiction, so common nowadays, to alleviate anguish and depression related to burnout.

Police forces in Mexico are facing huge challenges every day in a chaotic situation that endangers their life and puts them in a continuous state of worry which exacerbates their burnout. As shown in this study, policemen who exercise have lower rates of burnout development. Interventions should be applied with the purpose of daily activity as part of the policeman’s routine. Physical activity must become a mandatory requirement in their daily lives to stay healthy and socially involved and to improve their circle of trust and acceptance at the job site. Policemen in Mexico urgently need to be rescued from the daily nightmare in which they live: urgent attention to this topic needs to be paid and studies of this kind must be pushed to call the attention of the authorities and academics to the problem and its implications.

The main contributions to this research are the findings that show interventions and occupational health practices in the police force as a means of preventing worst case scenarios of burnout in Police Officers. This paper improves the understanding of burnout among policemen and the importance of exercise and leisure activities to alleviate burnout; therefore, serious attention should be paid to this matter that contributes to alleviate and reduce burnout syndrome and mental ill-health issues in this group of workers that are severely affected by their daily work routine.

In regard to gender differences, as shown in this research, there were significant differences between women’s and men’s burnout measurement. Research showed that, due to their double roles, traditional trends and responsibilities, women are at higher risk of burnout [39,61,62,63,64]. Therefore, women police officers; mental health risks should be monitored carefully and interventions involving sports and leisure activities should be included.

### 4.1. Future Opportunities for Research

From the results, it is recognized that similar research with larger samples is needed. Additionally, it is emphasized that there is a need to investigate the link between operational and organizational stressors in the prevalence of burnout syndrome in the professional activity of the police in future studies. There is still a need to investigate the relationship between burnout and physical and leisure activity in longitudinal studies. The individual and social impacts of burnout in policemen highlight the need for preventive interventions and early identification of this health condition in this environment. Therefore, future studies should deal with the specific efficacy of different exercise modalities and their combination, with further cognitive behavioral interventions. It would be interesting to study in greater depth the link between sports activity and burnout prevention. Further research is suggested in this regard, considering type and frequency of sports and impact on burnout.

### 4.2. Limitations and Strength of This Research

The main strength of the study was the evaluation of burnout syndrome occurrence in a population of police officers’ exposed to external extreme violence, as well as findings related to physical activities that were highly significant and positive. This opens opportunities for preventive interventions that can improve the quality of life of police officers and reduce negative impacts.

The limitations of this research were the sample size that could not be larger because of the small number of police officers working in the city at the time of the study. The answers to the questionnaire could have been influenced by fear of retaliation from their immediate supervisor, which raises worries about worse scenarios that could not be detected. Follow up of cases where addictions or severe health issues were detected was not possible due to the cross sectional study performed, and intervention was not part of the study.

## 5. Conclusions

To conclude, we have examined burnout syndrome and its relation with physical and leisure activities. The results showed that physical and leisure activities play an important role in the police officers well-being. These activities showed to have an impact in reducing negative emotions and promoting well-being. These findings should be taken into consideration by police corporations during the evaluation and assessment process to prevent policemen risk factors associated to burnout. Strategies that promote a healthier lifestyle and education programs about the benefits of regular physical exercise and leisure activities should be created. According to Norm 035-STPS-2018, programs that focus con occupational stress and burnout management should be implemented in police forces to allow the emotional discharge of tension build up by their labor activity. This study is expected to bring attention to the benefits of health promotion in the police forces. Interventive actions will help to reduce and alleviate psychological risk factors and disorders that are associated with burnout. New research should be focused on the effect of different types of physical and leisure activities related to burnout reduction.

## Figures and Tables

**Table 1:** Descriptive statistics for the SBI (Spanish Burnout Inventory) questions.

Items		Study Results					Figueiredo-Ferraz et al. (2014) [70] * Results					Gil-Monte & Noyola (2011) [69] ** Results			
		µ	σ	A	Corrected Total ρ	α without Item	μ	σ	A	Corrected Total ρ	α without Item	μ	σ	A	α without Item
Work Excitement (α = 0.64)							(α = 0.89)					(α = 0.72)			
item 1	Challenge work	2.39	(1.00)	−0.87	0.22	0.66	2.68	(0.92)	−0.68	0.49	0.88	3.55	(0.83)	−2.20	0.67
item 5	Personal fulfillment	3.15	(0.73)	−0.25	0.45	0.55	2.62	(1.11)	−0.40	0.55	0.86	3.74	(0.66)	−2.99	0.60
item 10	Positive things	2.74	(1.10)	−0.69	0.50	0.53	2.46	(1.03)	−0.37	0.51	0.87	3.51	(1.02)	−2.16	0.65
item 15	Gratifying job	3.09	(0.94)	−0.85	0.31	0.62	2.53	(1.18)	−0.56	0.64	0.85	3.75	(0.55)	−2.43	0.59
item 19	Excitement	2.28	(1.29)	−0.30	0.48	0.54	2.22	(1.14)	−0.25	0.59	0.86	3.36	(1.06)	−1.83	0.65
Mental Exhaustion (α = 0.85)							(α = 0.74)					(α = 0.86)			
item 8	Swamped	1.09	(0.91)	0.55	0.60	0.85	1.25	(1.02)	0.79	0.40	0.63	2.09	(1.22)	−0.12	0.86
item 12	Overwhelmed	0.70	(0.66)	0.43	0.71	0.80	1.37	(1.09)	0.59	0.31	0.70	1.35	(1.15)	0.46	0.80
item 17	Physical exhaustion	1.41	(1.15)	0.78	0.75	0.78	1.62	(1.06)	−0.35	0.29	0.68	1.39	(1.02)	0.41	0.79
item 18	Emotional exhaustion	1.22	(1.11)	0.76	0.72	0.80	1.80	(1.25)	0.21	0.27	0.70	1.09	(1.01)	0.85	0.81
Guilt (α = 0.79)							(α = 0.72)					(α = 0.79)			
item 4	Concern contact	0.72	(0.75)	0.52	0.37	0.83	1.28	(1.22)	0.77	0.34	0.66	1.02	(1.23)	1.33	0.78
item 9	Guilt-related attitudes	0.41	(0.54)	0.80	0.78	0.70	0.97	(0.99)	0.93	0.45	0.60	0.82	(0.98)	1.69	0.65
item 13	Remorse	0.30	(0.47)	0.88	0.78	0.70	0.72	(0.87)	1.22	0.38	0.63	0.51	(0.65)	1.32	0.69
item 16	Apologizing	0.61	(0.65)	0.60	0.59	0.75	0.83	(0.85)	1.26	0.34	0.65	0.67	(0.65)	0.85	0.74
item 20	Bad things said	0.48	(0.62)	0.95	0.48	0.78	1.25	(0.81)	0.53	0.03	0.78	0.60	(0.67)	0.86	0.69
Indolence (α = 0.71)							(α = 0.73)					(α = 0.75)			
item 2	Not assisting	0.57	(0.65)	0.74	0.37	0.69	1.45	(0.96)	0.28	0.32	0.66	1.03	(0.92)	0.94	0.67
item 3	Insufferable	1.11	(0.88)	0.82	0.56	0.62	1.65	(0.88)	0.35	0.27	0.70	0.87	(0.85)	1.08	0.68
item 6	Mean	1.09	(1.13)	0.98	0.46	0.67	1.16	(0.92)	0.61	0.33	0.66	0.58	(0.70)	1.12	0.68
item 7	Indifference	0.61	(0.68)	0.68	0.46	0.66	0.96	(1.00)	1.00	0.29	0.68	0.59	(0.64)	0.83	0.69
item 11	Irony	0.41	(0.54)	0.80	0.39	0.69	1.31	(1.00)	0.56	0.31	0.65	0.38	(0.83)	2.74	0.77
item 14	Labelling	1.09	(1.05)	0.78	0.47	0.66	1.55	(1.21)	0.42	0.11	0.75	0.83	(0.99)	1.44	0.72

* Results of the investigation of Figueiredo-Ferraz et al. (2014) [70]. ** Results of the investigation of Gil-Monte & Noyola (2011) [69]. **Source:** own elaboration from research results.

**Table 2:** Descriptive analysis and significance tests -ANOVA- of the attributes that form operational stressors for the two found clusters.

Items	Cluster 1 (*n* = 22)		Cluster 2 (*n* = 24)		Sig.	
	µ	σ	μ	σ		
Shift changes	2.50	(1.60)	4.46	(0.98)	0.000	***
Working the night shift	2.50	(1.65)	4.67	(2.28)	0.001	**
Request of overtime	2.82	(1.40)	5.13	(1.33)	0.000	***
Risk of being injured at work	2.36	(1.22)	4.75	(1.39)	0.000	***
Working in days off	3.95	(1.59)	5.71	(1.33)	0.000	***
Handling traumatic events	2.45	(1.41)	4.33	(1.34)	0.000	***
Insufficient time to spend with my family and friends	3.09	(1.48)	5.25	(1.54)	0.000	***
Too much paperwork	2.27	(1.12)	4.33	(1.74)	0.000	***

Cluster 1 presents occasional operational stressors. Cluster 2 presents daily operational stressors. Source: own elaboration from research results. *** 99% significance, ** 95% significance.

**Table 3:** Descriptive analysis and significance tests -ANOVA- of the attributes and the dimensions of CESQT for the two clusters found.

Descriptive Statistics	Cluster 1 (*n* = 60%)		Cluster 2 (*n* = 40%)		Sig.	
	μ	σ	μ	σ		
Challenge work	2.50	1.11	2.22	0.81	0.363	
Personal fulfillment	3.25	0.70	3.00	0.77	0.261	
Positive things	3.00	1.09	2.33	1.03	0.044	*
Gratifying job	3.32	0.90	2.72	0.89	0.033	*
Excitement	2.50	1.43	1.94	1.00	0.157	
Swamped	0.75	0.75	1.61	0.92	0.001	**
Overwhelmed	0.32	0.48	1.28	0.46	0.000	***
Physical exhaustion	1.00	1.02	2.06	1.06	0.002	**
Emotional exhaustion	0.64	0.78	2.11	0.96	0.000	***
Concern contact	0.43	0.63	1.17	0.71	0.001	**
Guilt-related attitudes	0.14	0.36	0.83	0.51	0.000	***
Remorse	0.07	0.26	0.67	0.49	0.000	***
Apologizing	0.36	0.49	1.00	0.69	0.001	**
Bad things said	0.29	0.53	0.78	0.65	0.007	**
Not answering	0.36	0.62	0.89	0.58	0.006	**
Insufferable	0.93	0.77	1.39	0.98	0.081	
Mean	0.64	0.73	1.78	1.31	0.000	***
Indifference	0.36	0.62	1.00	0.59	0.001	**
Irony	0.29	0.53	0.61	0.50	0.045	*
Labelling	0.93	1.05	1.33	1.03	0.206	
Work excitement	2.91	0.64	2.44	0.62	0.017	*
Psychical exhaustion	0.68	0.54	1.76	0.66	0.000	***
Guilt	0.26	0.30	0.89	0.35	0.000	***
Indolence	0.58	0.40	1.17	0.54	0.000	***
Burnout syndrome	1.11	0.29	1.57	0.35	0.000	***

Source: own elaboration from research results. * 90% significance, ** 95% significance, *** 99% significance.

**Table 4:** Analysis of variance (ANOVA) for the CESQT’s dimensions between the development of recreational activities, demographic conditions, and working conditions.

Dimension and Analysis Factor			Sum of Squares	DF *	Root Mean Square	F	Sig.
**H1.** Engaging in Sport Activities	Work excitement	Between groups	0.019	1	0.019	0.043	0.837
		Within groups	19.838	44	0.451		
		Total	19.857	45			
	Psychical exhaustion	Between groups	2.421	1	2.421	4.133	0.048
		Within groups	25.776	44	0.586		
		Total	28.197	45			
	Guilt	Between groups	0.195	1	0.195	0.99	0.325
		Within groups	8.664	44	0.197		
		Total	8.859	45			
	Indolence	Between groups	0.3	1	0.3	1.027	0.316
		Within groups	12.845	44	0.292		
		Total	13.145	45			
**H2.** Recreational Activities	Work excitement	Between groups	1.33	1	1.33	3.159	0.082
		Within groups	18.527	44	0.421		
		Total	19.857	45			
	Psychical exhaustion	Between groups	1.794	1	1.794	2.99	0.091
		Within groups	26.403	44	0.6		
		Total	28.197	45			
	Guilt	Between groups	0.082	1	0.082	0.41	0.525
		Within groups	8.777	44	0.199		
		Total	8.859	45			
	Indolence	Between groups	0.223	1	0.223	0.759	0.388
		Within groups	12.922	44	0.294		
		Total	13.145	45			
**H3.** Gender	Work excitement	Between groups	0.202	1	0.202	0.453	0.504
		Within groups	19.655	44	0.447		
		Total	19.857	45			
	Psychical exhaustion	Between groups	3.306	1	3.306	5.843	0.02
		Within groups	24.891	44	0.566		
		Total	28.197	45			
	Guilt	Between groups	0.141	1	0.141	0.711	0.404
		Within groups	8.718	44	0.198		
		Total	8.859	45			
	Indolence	Between groups	0.018	1	0.018	0.059	0.809
		Within groups	13.127	44	0.298		
		Total	13.145	45			
**H4.** Marital Status	Work excitement	Between groups	0.172	1	0.172	0.384	0.539
		Within groups	19.685	44	0.447		
		Total	19.857	45			
	Psychical exhaustion	Between groups	1.39	1	1.39	2.281	0.138
		Within groups	26.807	44	0.609		
		Total	28.197	45			
	Guilt	Between groups	0.27	1	0.27	1.384	0.246
		Within groups	8.589	44	0.195		
		Total	8.859	45			
	Indolence	Between groups	0.494	1	0.494	1.72	0.197
		Within groups	12.65	44	0.288		
		Total	13.145	45			
**H5.** Operational Stressors clusters	Work excitement	Between groups	0.748	1	0.748	1.723	0.196
		Within groups	19.109	44	0.434		
		Total	19.8573913	45			
	Psychical exhaustion	Between groups	3.427	1	3.427	6.087	0.018
		Within groups	24.770	44	0.563		
		Total	28.1970109	45			
	Guilt	Between groups	1.190	1	1.190	6.827	0.012
		Within groups	7.669	44	0.174		
		Total	8.85913043	45			
	Indolence	Between groups	0.356	1	0.356	1.225	0.274
		Within groups	12.789	44	0.291		
		Total	13.145	45			

Source: own elaboration from research results. * Degrees of Freedom.

**Table 5:** Hypotheses summary.

Hypothesis	Conclusion	Observations
H1: there are significant differences in the prevalence of burnout syndrome between police officers who engage in sport activities and those who do not.	Hypothesis H_1_ is accepted	It is emphasized that the hypothesis is accepted because engaging and not engaging in sport activities does show significant differences in the dimension of mental exhaustion (*p* value ≤ 0.05)
H2: there are significant differences in the prevalence of burnout syndrome between police officers who engage in recreational activities and those who do not.	Hypothesis H_2_ is rejected.	It is observed that developing recreational activities does not show significant differences in the prevalence of burnout syndrome (*p* value ≥ 0.05)
H3: there are significant differences in the prevalence of burnout syndrome between police officers in stable marriages and single officers.	Hypothesis H_3_ is rejected.	Marital status—whether or not having a stable partner—does not show significant differences in the prevalence of burnout syndrome (*p* value ≥ 0.05).
H4: there are significant differences in the prevalence of burnout syndrome between male and female professionals.	Hypothesis H_4_ is accepted.	There are significant differences between men and women in the dimension of mental exhaustion (*p* value ≤ 0.05)
H5: there are significant differences in the prevalence of burnout syndrome between police officers with daily operational stressors and police officers with occasional operational stressors.	Hypothesis H_5_ is accepted.	There are significant differences between the cluster with occasional operational stressors and the cluster with daily operational stressors in the dimensions of mental exhaustion and guilt (*p* value ≤ 0.05).

Source: Own elaboration.
